# *InsteaDMatic*: towards cross-platform automated continuous rotation electron diffraction

**DOI:** 10.1107/S1600576720009590

**Published:** 2020-08-20

**Authors:** Maria Roslova, Stef Smeets, Bin Wang, Thomas Thersleff, Hongyi Xu, Xiaodong Zou

**Affiliations:** a Department of Materials and Environmental Chemistry (MMK), Stockholm University, Svante Arrhenius väg 16C, Stockholm SE-10691, Sweden

**Keywords:** 3D electron diffraction, 3DED, microcrystal electron diffraction, microED, continuous rotation electron diffraction, cRED, automated data collection, *DigitalMicrograph* scripts, structure determination

## Abstract

A *DigitalMicrograph* script, *InsteaDMatic*, has been developed to facilitate rapid automated 3D electron diffraction/microcrystal electron diffraction data acquisition by continuous rotation of a crystal with a constant speed, denoted as continuous rotation electron diffraction. The script coordinates microscope functions, such as stage rotation, and camera functions relevant for data collection, and stores the experiment metadata. The script is compatible with any microscope that can be controlled by *DigitalMicrograph* and has been tested on both JEOL and Thermo Fisher Scientific microscopes. A proof of concept has been performed through employing *InsteaDMatic* for data collection and structure determination of a ZSM-5 zeolite. The influence of illumination settings and electron dose rate on the quality of diffraction data, unit-cell determination and structure solution has been investigated in order to optimize the data acquisition procedure.

## Abstract 2

A *DigitalMicrograph* script named *InsteaDMatic* has been developed for automated continuous rotation electron diffraction (cRED) data acquisition. *InsteaDMatic* coordinates functions of the transmission electron microscope goniometer and camera in order to tune up diffraction-frame recording simultaneously with crystal rotation. Exploiting fast and automated data collection, the influence of the electron dose rate on the quality of cRED data was studied, and structural models obtained for two different parallel-beam illumination modes (aperture selection and nanoprobe) were compared.

## 1. Introduction   

3D electron diffraction (3DED) and microcrystal electron diffraction (MicroED) have been shown to be powerful techniques for the structure determination of solids, and are especially advantageous for studies of micro- and nano­crystals. So far, hundreds of structures have been determined by 3DED (Gemmi *et al.*, 2019 ▸), including zeolites (Jiang *et al.*, 2011 ▸; Martínez-Franco *et al.*, 2013 ▸; Guo *et al.*, 2015 ▸; Simancas *et al.*, 2016 ▸; Lee *et al.*, 2018 ▸; Bieseki *et al.*, 2018 ▸; Zhang *et al.*, 2018 ▸; Smeets *et al.*, 2019 ▸), metal–organic frameworks (Denysenko *et al.*, 2011 ▸; Feyand *et al.*, 2012 ▸; Wang, Rhauderwiek *et al.*, 2018 ▸; Lenzen *et al.*, 2019 ▸), pharmaceuticals (van Genderen *et al.*, 2018 ▸; Gruene *et al.*, 2018 ▸; Jones *et al.*, 2018 ▸; Brázda *et al.*, 2019 ▸), proteins (Nannenga *et al.*, 2014 ▸; de la Cruz *et al.*, 2017 ▸; Xu *et al.*, 2019 ▸; Xu *et al.*, 2019 ▸; Lanza *et al.*, 2019 ▸) and many others.

Data collection by 3DED/MicroED was initially performed using a stepwise protocol, namely a set of electron diffraction patterns was recorded by tilting a crystal in fixed angular steps around an arbitrary crystallographic axis within the full range of the goniometer tilt (Kolb *et al.*, 2007 ▸; Zhang *et al.*, 2010 ▸; Shi *et al.*, 2013 ▸). Software packages dedicated to stepwise 3DED data collection and treatment were developed, known as automated diffraction tomography, *ADT* (Kolb *et al.*, 2007 ▸) and rotation electron diffraction, *RED* (Wan *et al.*, 2013 ▸). More recently, data collection by continuous rotation of a crystal at a constant speed was proposed by several groups (Nederlof *et al.*, 2013 ▸; Nannenga *et al.*, 2014 ▸; Gemmi *et al.*, 2015 ▸; Yonekura *et al.*, 2019 ▸), leading to the development of a technique known as continuous rotation electron diffraction (cRED). cRED is performed by recording ED frames while continuously rotating the crystal along a goniometer axis at a constant speed.

The basic hardware requirements for the transmission electron microscope (TEM) are only a single-tilt sample holder and a camera. Hence, data collection can be performed on a wide variety of TEMs. However, software control and synchronization of the TEM goniometer and the camera is required. Currently, only a limited number of software packages are designed to interface with both the camera and the microscope to collect multiple ED patterns simultaneously with crystal rotation. Many of them are commercial and/or closed source, *e.g. iTEM* from Olympus Soft Imaging Solutions (Gemmi *et al.*, 2015 ▸), *EPUd* (Thermo Fisher Scientific, 2019 ▸), *ParallEM* (Yonekura *et al.*, 2019 ▸) and *eTasED* (Zhou *et al.*, 2019 ▸). Recently, a script for *SerialEM* (Mastronarde, 2005 ▸), a widely used program in the cryo-electron microscopy community supporting electron microscopes and detectors from various manufacturers, has been used to enable large-scale MicroED data collection on Thermo Fisher Scientific (TFS) microscopes (de la Cruz *et al.*, 2019 ▸). Meanwhile, we have developed an open-source software platform, *Instamatic*, for electron crystallography needs, which is able to control both microscope and camera (Smeets *et al.*, 2018 ▸) and affords additional features such as crystal tracking through defocusing of the diffraction pattern (Cichocka *et al.*, 2018 ▸; Wang *et al.*, 2019 ▸). Automation of the data collection through *Instamatic* allows reproducible results to be collected with minimal human effort, especially for very large numbers of data sets. Currently, *Instamatic* is compatible with the Timepix detector (Amsterdam Scientific Instruments, The Netherlands) and the XF416/F416 cameras (Tietz Video and Image Processing Systems GmbH, Germany). However, additional developments are required for *Instamatic* to interface with other cameras. To the best of our knowledge, currently there is no flexible, cross-platform and easy-to-install software available for 3DED data collection. Many existing software packages are optimized only for the specific microscopes which are installed in the working groups developing the software. Therefore, it is highly desirable to develop software that can interface with and control a wide variety of cameras and microscopes made by different manufacturers, and ensure the hardware communications between them, even when they are controlled by separate computers. Such software should be easy to set up, straightforward to learn and user friendly.

Here, we propose to employ *DigitalMicrograph* (*DM*, Digital Micrograph Gatan, Pleasanton, California, USA) as a mediator controlling hardware interactions between the microscope and camera. We have developed a dedicated *DM* script, named *InsteaDMatic*, for automated cRED data collection. *InsteaDMatic* follows the same data collection workflow as described previously (Cichocka *et al.*, 2018 ▸) but communicates with both the microscope and camera *via* the *DM* interface. The benefit of this design philosophy is ease of installation and enhanced transferability, since the *DM* software is an integral part of a vast majority of electron microscopy systems nowadays. *InsteaDMatic* was first tested on our Themis Z (TFS) TEM equipped with a Gatan OneView IS camera and on a JEM2100F (JEOL) TEM with a Gatan Orius SC200D camera. Currently it has been successfully installed in more than ten other laboratories, equipped with various types of TEMs (JEM2100F, JEM3100F, Titan, Talos) and different cameras (Ultrascan, Orius, OneView). To demonstrate the capability of the script, we collected high-quality cRED data on a number of submicrometre-sized ZSM-5 zeolite crystals with up to 0.80 Å resolution, allowing accurate structure determination. The resulting data statistics were compared for crystals illuminated in selected-area mode and in parallel nanoprobe mode. To highlight the advantages of the approach, parameters such as electron dose rate and monochromator focus were tailored during the collection of cRED data.

## 2. Experimental   

### 2.1. Experimental setup   

The cRED experiments were performed on a Themis Z microscope equipped with a Gatan OneView IS camera (4096 × 4096 pixels, pixel size 15 µm) and a JEM 2100F TEM equipped with a Gatan Orius SC200D camera (2048 × 2048 pixels, pixel size 7.4 µm). The OneView camera is well suited for cRED data acquisition, because it has essentially no readout dead time when in movie mode. The *in situ* data capture mode with 1024 × 1024 pixel resolution (binning × 4) was employed. cRED data were collected using a single-tilt TFS holder (±40°) without applying a beam stopper. We found that the Themis Z is very stable both electrically and mechanically, and the crystal tracking procedure described by Cichocka *et al.* (2018 ▸) is not a prerequisite for keeping the crystal centred in the electron beam during data collection. Before data acquisition, a standard TEM alignment routine was performed. All experiments were performed in the parallel illumination mode using a 50 µm C2 condenser aperture. A suitable magnification (typically ×13 000) in the image mode at the SA magnification range is chosen to search for a suitable crystal. The crystal is then moved to the centre of the screen. In order to ensure the crystal stays in the area selected by the aperture or electron beam during crystal rotation, it is important to adjust the crystal height to the mechanical eucentric position of the goniometer. This is achieved by either enabling an α-wobbler (±15°) or manually tilting the goniometer and minimizing the crystal drift by changing the *Z* height of the crystal. Diffraction patterns were focused to obtain sharp spots in the diffraction mode. The rotation speed was 1.44° s^−1^ and the exposure time was 0.30 s per frame, leading to 0.432° per frame. A cRED data set with a total rotation range of ∼80° and 185 ED frames was collected in approximately 55 s.

Two different beam settings available on the Themis Z were tested, namely selected-area electron diffraction (SAED) and nanoprobe electron diffraction (NED) modes. In the SAED mode, a 40 µm SA aperture was inserted to limit the area used for diffraction, whereas in the NED mode the field of view was restricted by the beam size. Spot size 5 or 6 was usually used in the SAED mode, and spot size 11 in the NED mode. The electron dose on the specimen was controlled by varying the monochromator focus.

For the JEM 2100F equipped with a Gatan Orius SC200D detector (2048 × 2048 pixels, pixel size 7.4 µm), the exposure time and rotation speed were set up to be 0.5 s per frame and 0.444° s^−1^, leading to 0.222° per frame and resulting in 209 frames within the total rotation range of 46.42° collected in 104.5 s. The relatively small tilt range was due to the limit of the single-tilt holder for the microscope.

### 2.2. Data processing and structure determination   

Diffraction images were collected as TIFF files (.tif) and converted to SMV format (.img) using the *process_DM* Python script (Smeets, 2019 ▸). The collected frames were processed with the *XDS* software (Kabsch, 2010 ▸) for spot-finding, unit-cell determination, indexing, space-group assignment, data integration, scaling and refinement. The previously determined lattice parameters and space group (Olson *et al.*, 1981 ▸) were used as input, and the REFLECTING_RANGE_E.S.D. parameter in the XDS.INP file was set to be 0.7 to include very sharp diffraction spots in the indexing procedure. Data statistics indicators provided in the output CORRECT.Lp file were used for further data quality comparison. The reflection file for structure solution and refinement was obtained by merging several individual data sets from different crystals using the *XSCALE* sub-program. The structure was solved by *SHELXT* and refined by *SHELXL* (Sheldrick, 2008 ▸, 2015*b* ▸) using atomic structure factors for electrons (Doyle & Turner, 1968 ▸) with the help of the *OLEX2* software (Dolomanov *et al.*, 2009 ▸).

## 3. *InsteaDMatic* workflow   

*InsteaDMatic* follows the data collection workflow described by Cichocka *et al.* (2018 ▸) using the continuous rotation method for electron diffraction (Arndt & Wonacott, 1977 ▸; Nederlof *et al.*, 2013 ▸; Nannenga *et al.*, 2014 ▸; Gemmi *et al.*, 2015 ▸). The same workflow has previously been implemented in Python in the program *Instamatic* (Smeets *et al.*, 2018 ▸). However, *Instamatic* requires additional development to interface with different cameras.

**Figure 1:**
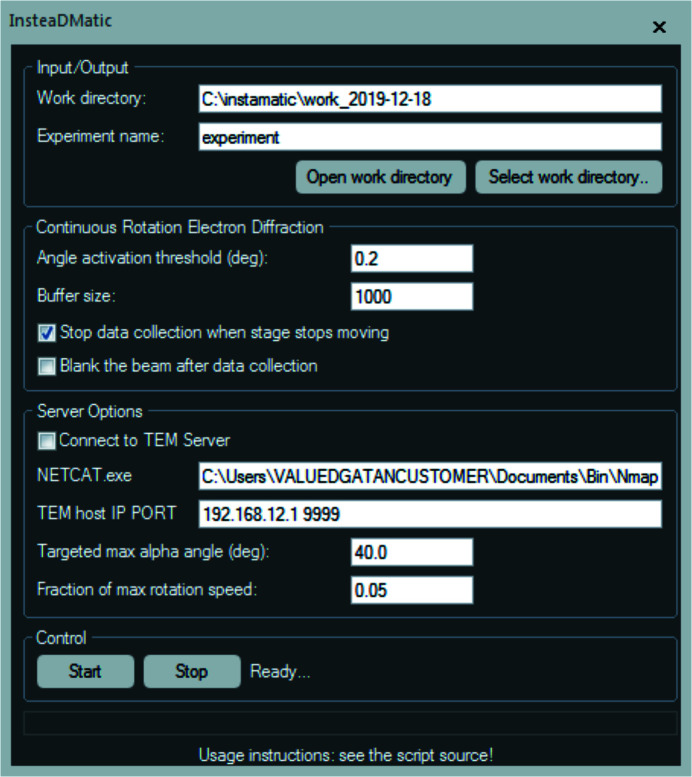
The graphical user interface of *InsteaDMatic*.

On the camera computer, *InsteaDMatic* is run from *DM* and the graphical user interface (GUI) is shown in Fig. 1 ▸. Settings for data collection (exposure, binning *etc.*) are defined through the camera panel in *DM*. When an experiment is started by pressing the ‘Start’ button at the very bottom of the GUI, the script enters a waiting state where it constantly polls the current α tilt value. Once a change larger than a pre-defined threshold (the angle activation threshold, typically 0.2°) is detected, data acquisition is initiated. The threshold also serves to eliminate any existing backlash in the α tilt direction. Rotation can then be initiated through any means available, either using the knobs, through the TEM user interface or using the software. At present, the *DM* API does not allow fine control over the rotation speed of the goniometer, although this function is available on our microscope (Themis Z, TFS), as well as other recent TFS/JEOL microscopes, through the *TEMScripting* interface. To be able to control the rotation through *DM*, we implemented a custom Python script in *Instamatic* (Smeets, 2018 ▸) to synchronize rotation with data acquisition. The script establishes an interface with the TEM on the microscope computer and accepts connections over the network. A socket interface is then established using the program *netcat* (https://nmap.org/ncat/) on the camera computer through the *DM* function *LaunchExternalProcess*, which then communicates the requested rotation range and speed over the network to the microscope computer. Once rotation has been detected, data acquisition is initiated. The *DM* script hooks into the live view of the OneView camera, and then constantly copies the front-most image to a pre-allocated ‘image buffer’ whose size can be defined in the GUI of the script (‘buffer size’) and corresponds to the maximum number of frames that are expected to be collected. Whenever the live view is updated, *DM* fires an event called DataValueChangedEvent, which signals the script to copy the frame. The exposure time and binning are therefore defined through the *DM* interface, and not through the script. Data collection may be interrupted at any time by pressing the ‘Stop’ button. There is also an automatic check for the completion of data collection, by monitoring the change in α tilt after every image cloning operation. When the change is equal to 0, the data collection loop breaks automatically. Finally, the script stores all relevant experimental metadata required for processing to a new directory, such as the rotation range, exposure time, camera length *etc*. The image files are stored in the same directory as TIFF files, and can be converted to other desired formats (SMV and MRC) by running the *process_DM.py* script (Smeets, 2019 ▸).

**Figure 2:**
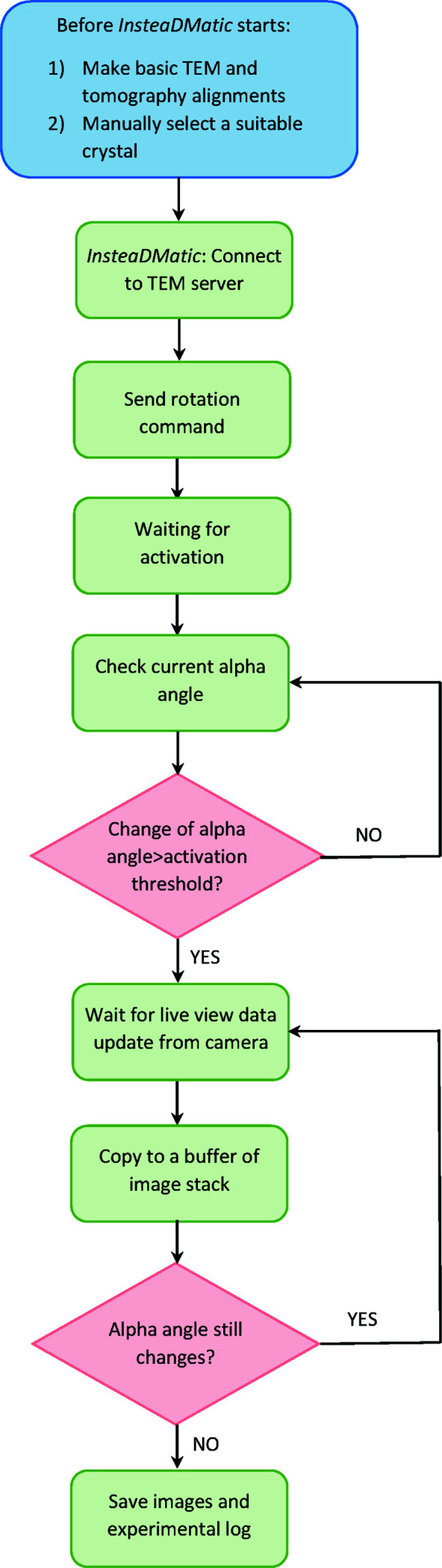
The workflow for 3DED data collection using *InsteaDMatic*. The blue box includes operations to be performed by the TEM operator, whereas the green and pink boxes show steps of the *InsteaDMatic* script protocol running automatically.

A flowchart of the workflow is shown in Fig. 2 ▸. The experimental procedure for a typical cRED data collection experiment is shown in the supporting information, Movie S1. Detailed instructions for usage can be found in the script. The script is compatible with *DM* Version 2.0 (which introduced the DataValueChangedEvent) or newer, and can be used with any Gatan camera that supports a streaming live view.

## 4. Application to structure determination of ZSM-5   

A proof of concept has been performed through employing *InsteaDMatic* for data collection and further structure determination of a ZSM-5 aluminosilicate zeolite widely used in industry as a catalyst (Choi *et al.*, 2009 ▸; Ji *et al.*, 2017 ▸). ZSM-5 is relatively stable against electron beam damage, allowing multiple data sets to be collected from the same crystal. Consequently, a direct comparison of cRED data quality at different illumination settings becomes possible. ZSM-5 was previously used as a test sample for the assessment of data quality and accuracy of structure determination by rotation electron diffraction (Su *et al.*, 2014 ▸), cRED (Wang, Yang *et al.*, 2018 ▸) and serial rotation electron diffraction (Wang *et al.*, 2019 ▸). For the cRED experiments, thoroughly ground ZSM-5 powder was dispersed in ethanol and then subjected to an ultrasonic bath treatment for 5 min. A drop of the suspension was applied to a lacey carbon grid (Cu150P from Okenshoji Co. Ltd, Japan) and dried in air for 10 min.

### 4.1. Tests of *InsteaDMatic* on Themis Z and JEM 2100F microscopes   

First, we tested *InsteaDMatic* on the Themis Z with a Gatan OneView CCD camera, collecting cRED data from different crystals. A typical experiment was recorded in order to illustrate the procedure of cRED data acquisition (see Movie S1). The best Themis Z data set demonstrated a completeness of 77.7% in the resolution shells ranging from 2.36 to 0.80 Å (see Table S1), enabling *ab initio* crystal structure solution from this one individual data set. Unfortunately, the completeness of most individual data sets does not exceed 50% for the orthorhombic structure, and often only merged data can provide the correct structure (see below).

We found that the OneView camera is well suited for experiments that require continuous read-out of the sensor. To check if the script would work on other cameras, we tested it on an Orius SC200D detector installed on a JEM 2100F. A ‘single-crystal’ data set collected over a rotation range of 46.42° reached a completeness of 34.5% in the resolution shells from 2.36 to 0.80 Å. Due to the limited tilting capability of the microscope, the data completeness is low, prohibiting a correct crystal structure solution by direct methods, *e.g. SIR2014* (Burla *et al.*, 2015 ▸) or *SHELXT* (Sheldrick, 2008 ▸, 2015*a ▸*).

### 4.2. cRED in SAED versus NED mode   

Traditionally, collection of electron diffraction data has been performed via diffraction area selection of a region of interest (ROI). However, the ROI selection can also be accomplished by adjusting the illumination settings. Almost parallel illumination with a sub-micrometre beam diameter can be obtained either by Köhler illumination (Wu *et al.*, 2004 ▸; Meyer *et al.*, 2006 ▸; Benner *et al.*, 2011 ▸) or by inserting a small C2 condenser aperture (Kolb *et al.*, 2007 ▸; Dwyer *et al.*, 2007 ▸). NED provides full control of the beam diameter and in principle allows collection of data on a smaller area than SAED (Gemmi *et al.*, 2019 ▸). However, in the literature there is a lack of direct comparison of data quality collected on the same sample by cRED in SAED and NED modes. Here, an attempt has been made to reveal the difference between these modes using the same area of the sample for collecting diffraction data.

In SAED mode, a diffraction field of about 750 nm was selected by inserting an SA. In NED mode, the beam was condensed to illuminate the 750 nm area, and the electron dose rate was kept equal to that in SAED mode by adjusting the monochromator focus. The two resulting data sets registered on the same isolated crystal are presented in Table 1 ▸.

**Figure 3:**
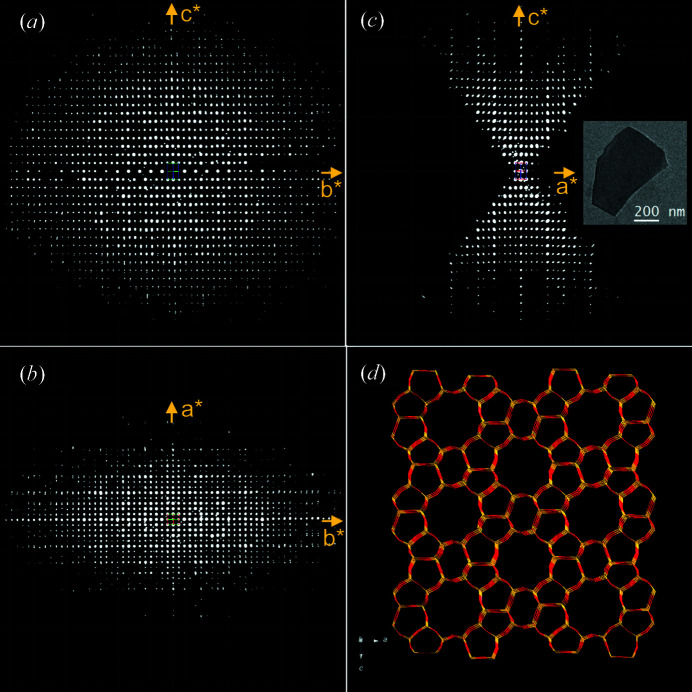
(*a*)–(*c*) Typical 3D reciprocal lattices of ZSM-5 reconstructed and visualized by *REDp* (Wan *et al.*, 2013 ▸). The corresponding crystal image is shown as an inset in (*b*). (*d*) A wire-and-stick ZSM-5 crystal structure representation.

Based on the previous crystallographic reports on the ZSM-5 single-crystal X-ray diffraction (SCXRD) structure (Olson *et al.*, 1981 ▸; van Koningsveld *et al.*, 1987 ▸), the lattice parameters *a* = 20.022 Å, *b* = 19.899 Å, *c* = 13.383 Å and the space group *Pnma* (No. 62) were used as input for *XDS*. Both SAED and NED data sets fit well with the expected orthorhombic structure and the refined unit-cell parameters are close to the published values within the accuracy of the 3DED method. Fig. 3 ▸ shows the reconstructed reciprocal lattice of ZSM-5 based on the cRED data collected in SAED mode from Table 1 ▸.

**Figure 4:**
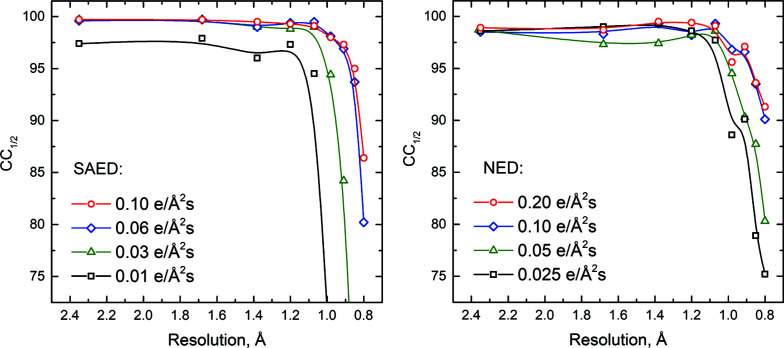
Effect of the electron dose rate on *CC*_1/2_ in different resolution shells. cRED data were collected with ROI selection either by selected-area aperture (SAED) or by nanoprobe illumination (NED). The ROI diameter was 750 nm. All SAED data were collected from the same ZSM-5 crystal sequentially, in ascending order of the electron dose rate. All NED data were collected from a second crystal following the same procedure. The lines in the figure are guides for the eye.

Among factors affecting the cRED data quality, electron dose has the utmost importance. Our experiments have shown that the optimal electron dose rate range for ZSM-5 data acquisition is approximately between 0.03 and 0.10 e Å^−2^ s^−1^ (Fig. 4 ▸). In the optimal range with no saturation, the higher the dose the better the *I*/σ. Excessive electron dose (>0.20 e Å^−2^ s^−1^) causes read-out biases of the OneView camera, whereas a low electron dose rate (<0.03 e Å^−2^ s^−1^) leads to significant deterioration of the signal-to-noise ratio and, as a consequence, to poor data statistics. Examples of the raw SAED/NED diffraction patterns collected at different electron doses are shown in Fig. S1. Since *XDS* relies upon the lowest measured intensities to guide subtraction of the background, the scaling of the Bragg intensities as a function of resolution shells unavoidably leads to significant deterioration of weak but still useful high-resolution signals, and consequently to higher *R* values in the high resolution shells (1.00–0.80 Å). For X-ray diffraction a common practice would be to truncate the data at the resolution at which the data still show correlations (indicated by * on the CC_1/2_ value) (Karplus & Diederichs, 2015 ▸). However, for electron diffraction, we found that including data out to a CC_1/2_ value of ∼70% leads to an improvement in the refined model.

Another important factor for data collection is the stability of the CompuStage, since currently *InsteaDMatic* does not provide an opportunity to track the continuously rotating crystal during data collection. We have shown that the specimen movement controlled by the CompuStage controller is smooth and the crystal does not move out of the beam, even without its position being realigned during cRED data acquisition. In a typical experiment, we observed a total drift of only a few nanometres for a 100 × 100 nm crystal rotated from −40 to 40°, accompanied by a jump of ∼50 nm at the beginning of the rotation (see Movie S2). It should be noted that crystal drift becomes more severe at high tilt angles and ED intensities are also commonly systematically disrupted by uncompensated *Z* height changes. It is therefore a better strategy to merge a number of data sets collected from different crystals in the range from −40 to 40°, instead of collecting cRED data from one or two crystals with a large tilt range, *e.g.* from −70 to 70°.

For the structure solution, five individual cRED data sets collected from different crystals in SAED mode were merged, chosen by performing hierarchical cluster analysis based on 15 data sets using an in-house-developed program called *edtools* (https://github.com/stefsmeets/edtools). For the NED data, six out of ten input cRED data sets were chosen for merging. Hierarchical cluster analysis helps to find structurally similar data with high correlation coefficients between scaled diffraction intensities and to reach high completeness by merging only few data sets (Wang *et al.*, 2019 ▸). However, it is worth noting that simple averaging of unit-cell parameters obtained from individual 3DED data sets may result in irrelevant interatomic distances in the final structure. Hence the unit-cell parameters of standard ZSM-5 (as-made ZSM-5, determined by SCXRD; van Koningsveld *et al.*, 1987 ▸) were used for the structure solution and refinement: see the International Zeolite Association Database of Zeolite Structures (http://www.iza-structure.org/databases/).

**Figure 5:**
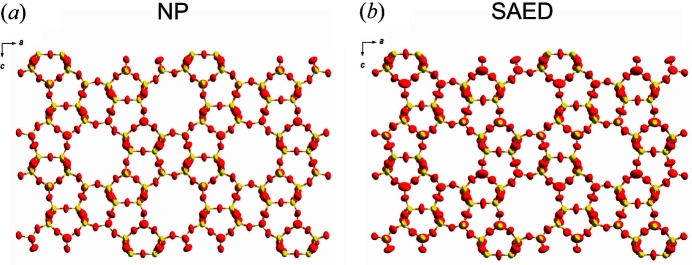
The framework structure of ZSM-5 viewed along the *b* axis as refined using (*a*) NED and (*b*) SAED data, showing anisotropic atomic displacement parameters for Si (yellow) and O (red) atoms. Displacement ellipsoids are drawn at the 50% probability level.

The structure of ZSM-5 can be solved using either *SIR2014* direct-space (Burla *et al.*, 2015 ▸) or *SHELXT* dual-space methods (Sheldrick, 2008 ▸). We note that a minimal *I*/σ signal-to-noise ratio of *ca* 2 (at 1.0 Å resolution limit) is required for revealing the framework of ZSM-5 by means of direct methods, whereas dual-space methods are not so sensitive to the *I*/σ ratio. There are 38 symmetry-independent atoms in the ZSM-5 structure, of which 12 are Si atoms and 26 are O atoms. There are four O atoms located at special positions. The atomic positions of all 12 Si and 26 O atoms were found successfully using both NED and SAED data, and used as an initial structural model. The details of the structure refinement are provided in Table 2 ▸. Anisotropic refinement of the NED model leads to *R*_1_ = 0.1758, goodness of fit (GoF) = 1.609, Si—O bond lengths in the range 1.555–1.635 Å and O—Si—O angles in the range 105.5–112.7°, with no additional restraints applied. For the SAED data, the refinement converged with *R*_1_ = 0.1992, GoF = 1.584, Si—O bond lengths in the range 1.551–1.635 Å and O—Si—O angles in the range 105.6–116.0°. Two restraints were applied to keep the Si—O bond lengths reasonable. In full agreement with the SCXRD model (Olson *et al.*, 1981 ▸; van Koningsveld *et al.*, 1987 ▸), the framework structure of ZSM-5 obtained from cRED data has a three-dimensional channel system with ten-ring straight channels of 5.4 × 5.6 Å in diameter running parallel to [010] and ten-ring sinusoidal channels of 5.1 × 5.4 Å in diameter running parallel to [100], as shown in Fig. 5 ▸.

A comparison with the reference model obtained from SCXRD (van Koningsveld *et al.*, 1987 ▸) was carried out using the *COMPSTRU* program (de la Flor *et al.*, 2016 ▸). All deviations of atomic positions between the reference ZSM-5 structure and those determined from cRED data are listed in Table S6. The deviations for the model obtained from the merged NED data set are on average 0.03 (1) Å for Si and 0.05 (2) Å for O, while those for the model obtained from the merged SAED data set are 0.05 (1) Å for Si and 0.07 (3) Å for O. This shows that cRED data collected using both NED and SAED provide reliable structural models. The NED data have a higher number of reflections with *I* > 2σ(*I*) (3903) than the SAED data (2854) (Table 2 ▸), which gives a slightly better structural model. The accuracy of the models is comparable to that obtained from our previous studies using single cRED data sets collected in SAED mode on a JEM-2100 LaB_6_ microscope equipped with a Timepix quad hybrid pixel detector (Wang, Yang *et al.*, 2018 ▸).

In contrast with the SAED mode, where the ROI to be used for data collection is pre-defined by the selected-area aperture size, the NED mode provides higher flexibility in adjusting the size of the area to be illuminated, and hence in fitting the size of each individual crystal so that the background in the ED frames is largely eliminated. This may be highly beneficial for studies of beam-sensitive materials since it paves the way for tailoring of the electron dose received by a specimen in a controllable manner.

## 5. Conclusions   

A new custom *DigitalMicrograph* script named *InsteaDMatic* has been developed to facilitate rapid automated 3DED/MicroED data acquisition using continuous rotation. *InsteaDMatic* has been successfully installed and operated on JEOL and Thermo Fisher Scientific microscopes utilizing *DigitalMicrograph* for control over the instrument and camera. The script was employed for data collection and structure determination of the ZSM-5 zeolite framework. A dose rate between 0.03 and 0.10 e Å^−2^ s^−1^ was found to be optimal for obtaining high-quality data with up to 0.80 Å resolution. The positions of the Si and O atoms in ZSM-5 can be found to within an accuracy better than 0.03 and 0.05 Å, respectively, from comparison with those obtained by SCXRD data. Both SAED and NED beam settings deliver an accurate structural model, provided that the beam and the stage are stable during goniometer rotation. Varying the monochromator focus offers an additional degree of freedom for tailoring the electron dose, which is especially relevant in the NED mode. We anticipate that the present research will contribute to the development of widely applicable routines for the structure determination of micro- and nanocrystals by 3DED/MicroED.

The *InsteaDMatic* script described in this article is available from https://github.com/stefsmeets/InsteaDMatic.

During preparation of this article, the *InsteaDMatic* script was successfully installed and tested in more than ten electron microscopy laboratories worldwide, and we gratefully acknowledge the feedback that we are receiving from them.

## Supplementary Material

Crystal structure: contains datablock(s) zsm5_cluster_3_saed, zsm5_cluster_1_np. DOI: 10.1107/S1600576720009590/te5059sup1.cif

Structure factors: contains datablock(s) zsm5_cluster_3_saed. DOI: 10.1107/S1600576720009590/te5059zsm5_cluster_3_saedsup2.hkl

Structure factors: contains datablock(s) zsm5_cluster_1_np. DOI: 10.1107/S1600576720009590/te5059zsm5_cluster_1_npsup3.hkl

Supporting Movie S1. Data acquisition workflow with InsteaDMatic. AVI format. DOI: 10.1107/S1600576720009590/te5059sup4.avi

Supporting Movie S2. Crystal drift during the cRED data acquisition. AVI format. DOI: 10.1107/S1600576720009590/te5059sup5.avi

Additional tables and figure. DOI: 10.1107/S1600576720009590/te5059sup6.pdf

CCDC references: 1998732, 1998733

## Figures and Tables

**Table 1:** Typical parameters for data collection and data processing of individual data sets by *XDS* Statistics in different resolution shells are given in Tables S2 and S3.

	SAED data set	NED data set
Spot size	5	11
Dose rate (e Å^−2^ s^−1^)	0.05	0.05
Diffraction area (nm)	750	750
Tilt range (°)	39.64 to −40.00	−39.71 to 40.00
Tilt step (°)	0.430	0.429
Exposure time (s)	0.30	0.30
Camera length (mm)	580	580
Mono focus	100.34	78.89
Rotation speed (° s^−1^)	1.441	1.434
Total No. of reflections	17224	17825
No. of unique reflections	2622	2692
Completeness (%)	47.1	48.3
Resolution cutoff (Å)	0.80	0.80
*I*/σ	4.19	4.42
*R*_obs_	0.208	0.217
*R*_exp_	0.239	0.248
*R*_meas_	0.228	0.239
*CC*_1/2_	98.7	98.1
Unit-cell parameters		
*a* (Å)	20.38 (4)	20.56 (4)
*b* (Å)	19.58 (1)	19.59 (1)
*c* (Å)	13.21 (2)	13.18 (2)

**Table 2:** Selected crystallographic data for merged ZSM-5 data sets Space group *Pnma* (No. 62), unit-cell parameters *a* = 20.022 (4) Å, *b* = 19.899 (4) Å, *c* = 13.383 (3) Å, electron wavelength λ = 0.0197 Å. Statistics in different resolution shells are given in Tables S4 and S5.

	SAED	NED
Data sets merged	5	6
Total No. of reflections	61596	65672
No. of unique reflections	5159	5299
No. of reflections with *I* > 2σ(*I*)	2854	3903
*R*_int_	0.3082	0.2282
Completeness (%)	95.8	98.2
Resolution cutoff (Å)	0.80	0.80
No. of parameters	332	332
No. of restraints	2	0
*R*_1_ [*I* > 2σ(*I*)]	0.1992	0.1758
*R*_1_ (all data)	0.2612	0.1997
GoF	1.584	1.609
