# Supplementary material for: InsteaDMatic: towards cross-platform automated continuous rotation electron diffraction
Source: J Appl Crystallogr. 2020 Aug 20;53(Pt 5):1217–24. doi: 10.1107/S1600576720009590 (PMC7534539; doi:10.1107/S1600576720009590)

JOURNAL OF
APPLIED
CRYSTALLOGRAPHY

Volume 53 (2020)

Supporting information for article:

InsteaDMatic: towards cross-platform automated continuous rotation electron diffraction

Maria Roslova, Stef Smeets, Bin Wang, Thomas Thersleff, Hongyi Xu and Xiaodong Zou

Table S1 Comparison of cRED data from Themis Z with One View camera and JEM2100F with Orius SC200D camera.

	Themis Z / One View	JEOL2100F / Orius SC200D
Spot size	5	1
Dose rate, e/Å^{2}s	0.05	0.084
Diffraction area, nm	750	1200
Tilt range, °	29.77 to -29.99	-22.48 to 23.94
Oscillation angle, °	0.424	0.222
Exposure time, s	0.30	0.50
Acquisition time per frame, s	0.30	0.50
Camera length, mm	580	800
Rotation speed, °/s	1.421	0.444
Rotation axis, °	-171	-42.5
Total No. of reflections	12055	11042
No. of unique reflections	4276	2086
Completeness, %	77.7	34.5
Resolution cutoff, Å	0.80	0.80
I/σ	4.19	3.08
R_{obs}, %	11.0	23.3
R_{exp}, %	12.4	30.0
R_{meas}, %	13.7	26.1
$CC_{1/2}$	99.1	98.6
Unit cell parameters		
a/Å	19.93(2)	20.68(2)
b/Å	19.48(3)	20.49(2)
c/Å	13.461(9)	13.75(1)

Table S2 Statistics of an individual cRED dataset collected in SAED mode on Themis Z in different resolution shells.

Resolution limit	#obs	#uniq	#pos	comp %	R_{obs} %	R_{exp} %	#comp	I/σ	R_{meas} %	$CC_{1/2}$
2.35	605	107	233	45.9	12.2	13.2	601	10.91	13.5	98.3
1.68	1101	181	382	47.4	16.0	14.9	1097	8.24	17.6	98.0
1.38	1460	233	496	47.0	17.0	16.0	1452	7.41	18.7	98.5
1.20	1789	276	574	48.1	19.2	19.1	1785	6.27	21.2	97.9
1.07	2103	312	650	48.0	21.8	22.0	2098	5.47	23.7	97.9
0.98	2315	344	719	47.8	43.8	52.1	2300	3.09	47.6	93.9
0.91	2502	374	786	47.6	48.0	72.4	2489	2.51	52.2	92.5
0.85	2724	400	832	48.1	70.5	117.0	2714	1.71	76.5	90.1
0.80	2625	395	890	44.4	89.3	173.9	2614	1.21	97.0	88.7
total	17224	2622	5562	47.1	20.8	23.9	17150	4.19	22.8	98.5

Table S3 Statistics of an individual cRED dataset collected in NED mode on Themis Z in different resolution shells.

Resolution limit	#obs	#uniq	#pos	comp %	R_{obs} %	R_{exp} %	#comp	I/σ	R_{meas} %	$CC_{1/2}$
2.36	653	112	234	47.9	13.2	15.6	651	9.74	14.6	98.7
1.68	1149	182	384	47.4	17.5	16.8	1146	8.26	19.2	97.3
1.38	1500	239	490	48.8	21.4	18.3	1495	7.15	23.5	95.4
1.20	1845	275	578	47.6	22.4	21.0	1843	6.61	24.8	98.2
1.07	2092	319	649	49.2	23.0	22.9	2079	5.71	25.1	98.6
0.98	2382	351	721	48.7	40.9	55.0	2376	3.48	44.8	94.5
0.91	2600	380	789	48.2	44.8	69.2	2595	3.10	49.1	90.3
0.85	2782	409	829	49.3	57.6	101.3	2767	2.25	62.9	90.7
0.80	2822	425	899	47.3	65.2	144.0	2810	1.49	71.1	93.7
total	17825	2692	5573	48.3	21.7	24.8	17762	4.42	23.9	98.1

Table S4 Statistics of SAED dataset merged from 5 crystals in different resolution shells.

Resolution limit	#obs	#uniq	#pos	comp %	R_{obs} %	R_{exp} %	#comp	I/σ	R_{meas} %	$CC_{1/2}$
3.58	571	61	67	91.0	21.8	27.3	571	7.64	23.4	94.2
2.53	1051	104	110	94.5	25.4	27.1	1051	7.35	26.7	95.5
2.07	1479	143	150	95.3	29.5	27.7	1479	6.91	31.2	97.4
1.79	1796	164	175	93.7	23.7	27.7	1796	6.77	25.1	98.2
1.60	1927	179	189	94.7	32.6	28.6	1927	6.06	34.6	88.9
1.46	2228	201	208	96.6	32.7	30.5	2226	5.71	34.3	98.4
1.35	2641	233	241	96.7	34.1	29.9	2640	5.22	36.0	94.3
1.26	2755	238	244	97.5	50.8	33.7	2752	4.99	53.5	81.3
1.19	2995	256	267	95.9	34.2	33.0	2994	5.15	36.1	93.8
1.13	3165	269	277	97.1	40.7	35.2	3163	4.85	42.7	93.4
1.08	3373	278	289	96.2	48.2	43.5	3370	4.07	50.8	90.9
0.99	3883	304	318	95.6	93.8	101.8	3881	2.82	98.0	89.1
0.92	4093	327	334	97.9	113.9	131.4	4089	2.45	119.2	86.6
0.89	4500	350	366	95.6	134.7	160.6	4498	2.30	140.6	86.2
0.84	4823	371	380	97.6	251.8	337.5	4819	1.74	262.5	61.4
0.82	4732	366	381	96.1	296.4	405.5	4729	1.53	309.0	55.8
0.80	3357	337	390	86.4	255.0	394.5	3347	1.35	267.6	69.2
total	61596	5159	5386	95.8	33.0	33.8	61551	3.56	34.9	94.8

Table S5 Statistics of NED dataset merged from 6 crystals in different resolution shells.

Resolution limit	#obs	#uniq	#pos	comp %	R_{obs} %	R_{exp} %	#comp	I/σ	R_{meas} %	$CC_{1/2}$
3.58	705	67	68	98.5	20.0	23.6	705	9.53	21.2	98.0
2.53	1302	112	112	100.0	17.0	24.0	1302	9.00	17.9	98.5
2.07	1775	146	146	100.0	19.6	24.5	1775	8.59	20.6	97.7
1.79	2188	175	175	100.0	22.3	24.8	2188	7.81	23.6	97.5
1.60	2507	196	196	100.0	21.4	25.9	2507	7.44	22.5	98.5
1.46	2690	203	205	99.0	29.8	27.4	2690	6.98	31.2	99.6
1.35	3066	232	233	99.6	23.4	26.5	3066	6.84	24.6	84.3
1.27	3422	255	255	100.0	28.2	29.5	3422	6.13	29.5	98.9
1.19	3528	258	258	100.0	26.2	29.6	3528	6.38	27.4	98.8
1.13	3881	287	288	99.7	27.7	30.7	3881	5.83	28.9	96.6
1.08	4016	283	285	99.3	38.8	39.1	4016	5.22	40.4	98.8
0.99	3804	324	325	99.7	53.3	51.6	3804	3.73	55.9	95.3
0.92	4069	341	341	100.0	54.4	56.9	4069	3.41	57.1	92.7
0.89	4268	358	358	100.0	67.0	65.6	4268	2.89	70.1	92.3
0.84	4731	395	395	100.0	90.4	103.9	4731	2.27	94.7	84.3
0.82	4490	383	388	98.7	91.6	114.8	4490	2.18	95.9	95.7
0.80	3234	310	394	78.7	91.8	112.8	3227	1.83	96.6	83.7
total	65672	5299	5397	98.2	24.0	27.7	65665	4.56	25.2	97.4

Table S6 Deviations of atomic positions between the reference ZSM-5 structure (van Koningsveld *et al.*, 1987) and those determined from cRED data collected in SAED/NED modes. Fractional atomic coordinates for the reference ZSM-5 structure determined by SCXRD are given in Table S7 (as-made ZSM-5, space group *Pnma*, a = 20.022(4) Å, b = 19.899(4) Å, c = 13.383(3) Å, see the International Zeolite Association (IZA) Database).

Atom	Atomic displacement SAED, Å	Atomic displacement NED, Å
Si1	0.064	0.027
Si2	0.063	0.042
Si3	0.043	0.032
Si4	0.021	0.027
Si5	0.021	0.021
Si6	0.063	0.007
Si7	0.046	0.014
Si8	0.051	0.022
Si9	0.053	0.030
Si10	0.051	0.036
Si11	0.055	0.035
Si12	0.044	0.016
O1	0.074	0.050
O2	0.105	0.053
O3	0.022	0.037
O4	0.067	0.051
O5	0.032	0.028
O6	0.066	0.025
O7	0.070	0.001
O8	0.053	0.029
O9	0.041	0.026
O10	0.101	0.082
O11	0.011	0.047
O12	0.122	0.124
O13	0.098	0.073
O14	0.097	0.032
O15	0.075	0.060
O16	0.046	0.028
O17	0.081	0.048
O18	0.095	0.049
O19	0.070	0.043
O20	0.045	0.030
O21	0.072	0.036
O22	0.023	0.073
O23	0.070	0.080
O24	0.068	0.060
O25	0.075	0.065
O26	0.092	0.029
**<Si> average**	**0.05(2)**	**0.03(1)**
**<O> average**	**0.07(3)**	**0.05(2)**

Table S7 Fractional atomic coordinates for the reference ZSM-5 structure (van Koningsveld *et al.*, 1987).

Atom	*x*	*y*	*z*
Si1	0.4224	0.0565	−0.3360
Si2	0.3072	0.0277	−0.1893
Si3	0.2791	0.0613	0.0312
Si4	0.1221	0.0630	0.0267
Si5	0.0713	0.0272	−0.1855
Si6	0.1864	0.0590	−0.3282
Si7	0.4227	−0.1725	−0.3272
Si8	0.3078	−0.1302	−0.1855
Si9	0.2755	−0.1728	0.0311
Si10	0.1206	−0.1731	0.0298
Si11	0.0704	−0.1304	−0.1820
Si12	0.1871	−0.1733	−0.3193
O1	0.3726	0.0534	−0.2442
O2	0.3084	0.0587	−0.0789
O3	0.2007	0.0592	0.0289
O4	0.0969	0.0611	−0.0856
O5	0.1149	0.0541	−0.2763
O6	0.2435	0.0553	−0.2460
O7	0.3742	−0.1561	−0.2372
O8	0.3085	−0.1552	−0.0728
O9	0.1980	−0.1554	0.0288
O10	0.0910	−0.1614	−0.0777
O11	0.1169	−0.1578	−0.2694
O12	0.2448	−0.1594	−0.2422
O13	0.3047	−0.0510	−0.1866
O14	0.0768	−0.0519	−0.1769
O15	0.4161	0.1276	−0.3896
O16	0.4086	−0.0017	−0.4136
O17	0.4020	−0.1314	−0.4239
O18	0.1886	0.1298	−0.3836
O19	0.1940	0.0007	−0.4082
O20	0.1951	−0.1291	−0.4190
O21	−0.0037	0.0502	−0.2080
O22	−0.0040	−0.1528	−0.2078
O23	0.4192	−0.2500	−0.3540
O24	0.1884	−0.2500	−0.3538
O25	0.2883	−0.2500	0.0579
O26	0.1085	−0.2500	0.0611

Figure S1 Typical diffraction patterns of ZSM-5 collected on Themis Z / One View at different electron dose rates. Dotted rings indicate 0.8 Å resolution.

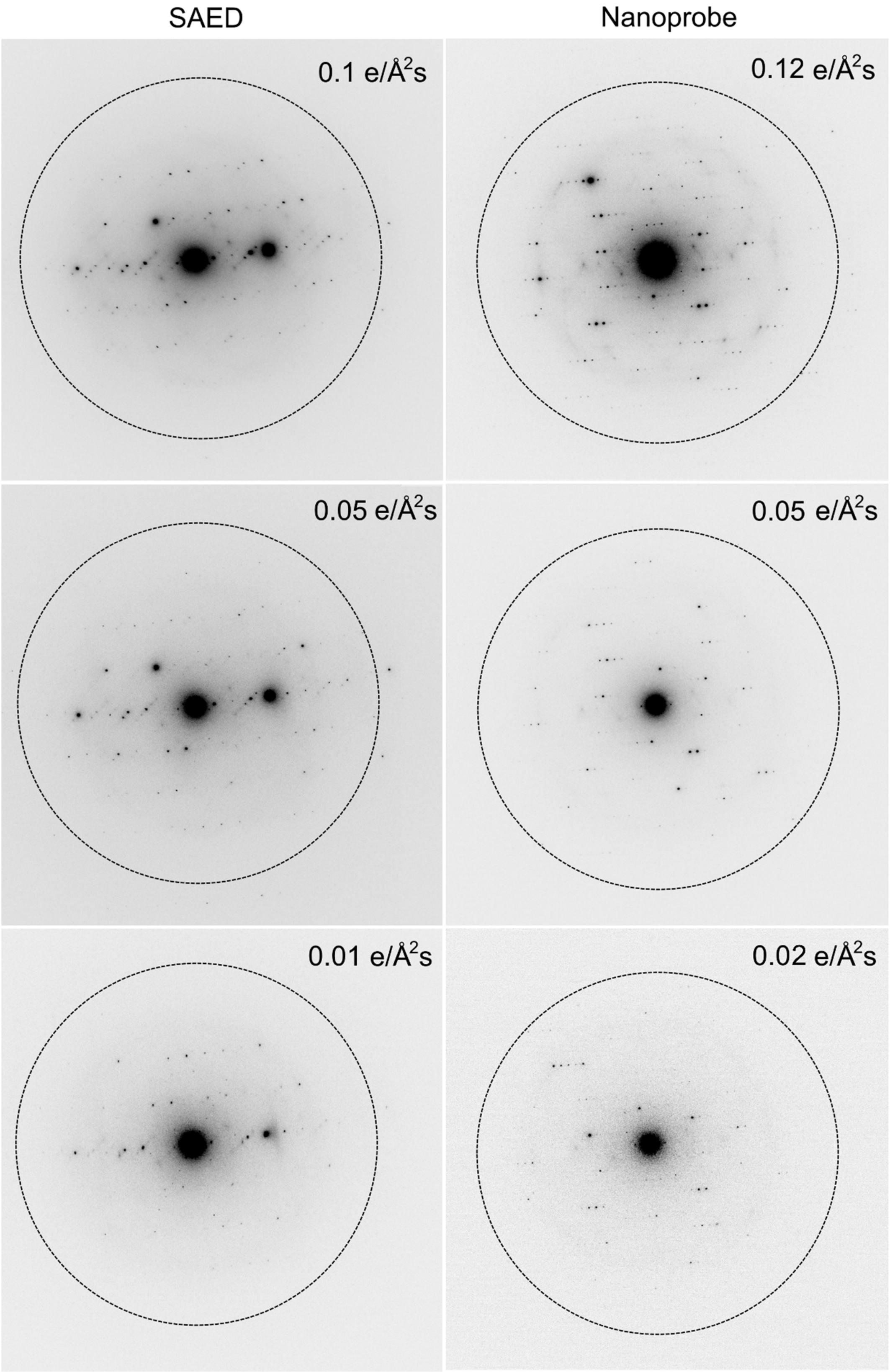

Supplement: Supplementary file 6 [file j-53-01217-sup6.pdf]
